# The Office, Duties, and Qualifications of Coroners

**Published:** 1839-04

**Authors:** 


PART FOURTH.

### Medical Intelligence.

### THE OFFICE, DUTIES, AND QUALIFICATIONS OF CORONERS.

THE Coroner is an ancient officer in England. He is called *coroner* because he had principally to do with pleas of *the crown*, or such wherein the King was more immediately concerned. In this respect the Lord Chief Justice of England is the principal coroner, and may, if he please, exercise the jurisdiction of a coroner in any part of the realm. There are also particular coroners for every county in England, in some counties two, and in others a greater number. This officer is of equal authority with *the sheriff*, and was ordained, together with him, to keep the peace when the earls gave up the wardship of the counties. Through the culpable neglect of gentlemen of property, this office has, in later times, been suffered to fall into disrepute, and get into low and indigent hands. Although formerly no coroners were paid, and by the statute of Westminster 1, they were expressly forbidden to take a reward, yet for many years past they have only desired to be chosen for the sake of the perquisites, being first allowed fees for their attendance by the 3 Henry VII., c. 1, of which Sir Edward Coke complained heavily. Since his time, coroners' fees have been much enlarged. It is unnecessary for us to inform the public that until large fees became attached to the office of coroner, *lawyers* were not wont to crave election to it.

Coroners for *counties* (except where custom to the contrary prevails) are chosen by all the freeholders of the county. Formerly the county members of parliament were chosen by the same electors. Now, however, by the Reform Act, the franchise, as respects the election of county members, has been somewhat altered; other interests than *freehold* confer the right of voting for members for counties. The Reform Act having extended the electors of members for counties, it becomes irreconcilable that the election of coroners for counties should be by the *freeholders only*. The election for a coroner of any county renders it matter of duty, at least with the freeholders of that county, to consider whether it be indispensable that the gentleman to be elected should be of the legal profession; if it be indispensable that a coroner should be a *lawyer*, it would become the duty of the county freeholders to vote against the medical candidate, for they are bound not to elect an inefficient officer; but it by no means follows that in the election of a barrister, or an attorney, a *lawyer* would thus be procured to fill the office. But very dangerous to the public welfare would it be, also, if lawyers *only* were deemed eligible for appointment to all offices connected with the administration of *justice*.

The peculiar duty of a coroner, the only one to which *especial* regard is had upon occasion of appointing him to his office, is to enquire, by a jury over whom he presides, concerning the manner of death when any person is slain, or dies suddenly, or in prison, within the circuit of his jurisdiction. Public decency requires that in all such cases the coroner should hold his enquiry with all possible speed; tenderness to the feelings of survivors prompts that such enquiry should not be unnecessarily prolonged. The manner of holding an inquest is peculiar, and perhaps unavoidably so: the constable of the district, so to speak, scrambles the witnesses together, and every one who imagines he has a tale to tell, either hastens to the coroner's court to offer it in evidence, or he keeps out of the way for a purpose of an opposite nature. It not unfrequently happens, that upon enquiries of this description no person appears to take part in the investigation; sometimes no witnesses are in attendance, except such as the constable has *in his wisdom* summoned.

It is manifest that in a court so convened, offering such imperfect assurance that the required witnesses are in attendance to give evidence, the witnesses cannot be held to that strict line of examination observed in superior courts, without injury to public justice. On the contrary, justice seems rather to require that some latitude should be allowed to witnesses in offering testimony before such a tribunal. If this be so, and it is impossible to doubt the fact, who, we ask, can be so unfit for coroner as a crotchety and, perchance, legally speaking, a half-educated *attorney?* His strictness would be out of place and season, and tend rather to stifle than develop the truth; whereas, a *non-legal* gentleman, proceeding upon the enquiry in an intelligent, matter of-fact manner, would not let one feature escape him, in however undigested or irregular a form it might be presented to himself and the jury. If it be true that a gentleman of education can ably fill the the office of coroner, although not of the legal profession, it follows that *a medical gentleman is the most proper man for the office;* for can it be denied that, upon view of the body, he would approach his subject with an intelligence which would be of unspeakable advantage to public justice, almost forbidding the possibility of concealment in any case where death has resulted by other than natural causes?

*Lancet. March* 9, 1839.

[Our principal object in reprinting this note respecting Coroners and the Coronership, is to call the attention of our readers to a subject of great importance not only to medical men but to the community generally. The recent triumphant election of Mr. Wakley to the coronership of Middlesex, will give *action* to the opinion generally prevailing among the members of our profession, that this office should be always filled by a medical man,—or, at least, by a man possessing a competent degree of medical knowledge. In this opinion we entirely concur; and recommend our brethren to lose no opportunity of endeavouring to obtain coronerships in towns and counties, as they become vacant. We must, however, remind them that it is very far from being true that every medical man is qualified for being a coroner; on the contrary, we believe that mere medical knowledge, however extensive or accurate,—without some general knowledge of law, and familiarity with the practice of courts,—is almost as inadequate qualification for the office as was the mere law without any medical knowledge, according to the old system.]

